# Construction of a regression model and post-procedure care strategy for predicting atrial fibrillation recurrence risk after radiofrequency ablation using combined P-wave electrocardiographic markers and serum

**DOI:** 10.3389/fcvm.2026.1733008

**Published:** 2026-05-13

**Authors:** Dong Zhao, Yahong Chen, Hongwei Zhang, Lixia Yang

**Affiliations:** 1 Interventional Operating Room, China-Japan Union Hospital of Jilin University, Changchun, China; 2 Department of Vascular Surgery, China-Japan Union Hospital of Jilin University, Changchun, China

**Keywords:** atrial fibrillation, brain natriuretic peptide, nomogram, P-wave ECG parameters, radiofrequency ablation, recurrence

## Abstract

**Background:**

To construct a risk prediction nomogram combining P-wave electrocardiographic indicators with serum brain natriuretic peptide (BNP) levels for predicting recurrence risk after radiofrequency ablation (RFA) for atrial fibrillation (AF), and to propose follow-up care strategies, thereby providing a theoretical basis for clinical prevention of early AF recurrence after RFA.

**Methods:**

A retrospective study was conducted on 200 AF patients who underwent radiofrequency ablation at our hospital between March 2023 and December 2024. All patients were confirmed to be in sinus rhythm at the time of preoperative electrocardiographic recording. Based on recurrence status at 3-month follow-up, patients were divided into recurrence (*n* = 62) and non-recurrence (*n* = 138) groups. Recurrence was assessed through a standardized rhythm monitoring protocol including scheduled electrocardiograms, 24 h Holter monitoring, and symptom-driven event recordings. The last enrolled patient completed follow-up on March 31, 2025.

**Results:**

Comparisons of AF type, left atrial diameter (LAD), P-wave duration (PWD), maximum P-wave duration (Pmax), P-wave dispersion (Pd), and BNP levels between groups showed statistically significant differences (*P* < 0.05). Logistic regression analysis confirmed that AF type, LAD, PWD, Pmax, Pd, and BNP were independent risk factors for post-AF radiofrequency ablation recurrence (OR > 1, *P* < 0.05). A risk prediction nomogram model was constructed. In the derivation cohort, the area under the receiver operating characteristic curve (AUC) was 0.959 (95% CI: 0.925–0.993). Apparent calibration was acceptable (Hosmer-Lemeshow test *P* = 0.726). Risk stratification analysis identified a total nomogram score of 85 points as an optimal threshold to distinguish low-risk from medium-to-high-risk patients. Formal internal validation was not performed.

**Conclusion:**

The derivation-cohort risk prediction nomogram based on P-wave ECG indicators combined with serum BNP demonstrates potential value for estimating early recurrence after AF radiofrequency ablation. Clinicians should interpret the model cautiously and develop personalized follow-up care strategies based on individual risk profiles to reduce recurrence risk and improve patient quality of life.

## 1 Introduction

Atrial fibrillation (AF) is a common sustained arrhythmia characterized by disorganized atrial electrical activity leading to ineffective atrial contraction (1). Epidemiological data indicate that AF prevalence increases substantially with age, reaching approximately 12% in individuals aged 75–84 years and as high as 33.3% among those over 84 years (2). The median age of AF patients is approximately 75 years, with individuals aged 65–85 accounting for 70% and those over 80 years representing one-third of the total patient population (3). AF episodes commonly present with palpitations, dizziness, and chest tightness, while severe cases may develop syncope, angina pectoris, or acute pulmonary edema. Elderly patients face an elevated risk of stroke, heart failure, myocardial infarction, cognitive decline, and renal impairment, posing substantial threats to health and increasing socioeconomic and healthcare burdens (4).

Contemporary AF management encompasses three principal therapeutic strategies: anticoagulation therapy for thromboembolic prevention, rate control, and treatments to restore and maintain sinus rhythm (5). Catheter-based radiofrequency ablation (RFA) is currently recognized as an effective rhythm-control strategy for AF, capable of maintaining sinus rhythm and improving symptoms, and is recommended as a therapeutic option in current guidelines (6). Despite continuous advances in ablation technology and improved procedural success rates, postoperative recurrence remains a significant clinical challenge, with reported incidence rates of approximately 20%–50% depending on AF type and follow-up duration (7).

Recent studies indicate that AF is associated with atrial structural and electrical remodeling. Electrocardiographic P-wave parameters reflect the severity of atrial pathology and may predict AF outcomes, though measurement variability related to individual characteristics and operator proficiency warrants consideration (8). Brain natriuretic peptide (BNP), a cardiac peptide reflecting myocardial ischemia, injury, and ventricular wall pressure, shows elevated expression during AF episodes (9). However, the relationship between BNP and postoperative AF recurrence, along with its predictive value, requires further validation. Notably, patients returning home after AF procedures may exhibit early signs of recurrence, such as blood pressure changes or arrhythmias, and failure to intervene promptly may adversely affect prognosis (10). In this context, continuity of care—the provision of ongoing, holistic nursing services by medical teams and social support systems after patient discharge—assumes particular relevance (11). When integrated with reliable risk prediction tools such as nomogram-based risk stratification, continuity of care enables the early detection and targeted management of patients at elevated recurrence risk, thereby facilitating timely intervention and improving postoperative outcomes.

Currently, systematic research combining the predictive value of P-wave ECG indicators and serum BNP for post-AF radiofrequency ablation recurrence with follow-up care measures is limited. This retrospective cohort study developed a risk prediction nomogram combining P-wave ECG indicators with serum BNP for estimating early recurrence after AF radiofrequency ablation. It also proposes guideline-aligned follow-up care strategies to provide clinicians with a clinically interpretable derivation model and a theoretical foundation for developing care protocols.

## 2 Materials and methods

### 2.1 Ethical statement

This study was approved by the Ethics Committee of China-Japan Union Hospital of Jilin University. Given its retrospective nature and use of de-identified patient data, informed consent was not required as no risk or detriment to patients was anticipated. This exemption complies with regulations and ethical guidelines pertaining to retrospective studies.

### 2.2 Study design

This retrospective study included 200 AF patients who underwent radiofrequency ablation at our hospital between March 2023 and December 2024. All patient data were extracted from the electronic medical record system. Based on recurrence status at the 3-month postoperative follow-up, patients were categorized into a recurrence group (*n* = 62) and a non-recurrence group (*n* = 138). To ensure complete ascertainment of the primary endpoint for all enrolled patients, follow-up continued until March 31, 2025.

### 2.3 Inclusion and exclusion criteria

Inclusion criteria were as follows: (1) clinically confirmed AF by electrocardiogram, primarily manifested as f-waves of varying amplitude and frequency replacing normal P-waves and absolute irregularity of RR intervals; (2) age ≥ 18 years; (3) documented sinus rhythm at the time of preoperative ECG recording, irrespective of AF subtype, to ensure the validity of P-wave measurements; (4) clear indications for radiofrequency ablation with no relevant contraindications. Early recurrence of AF was defined as electrocardiographically documented atrial fibrillation, atrial flutter, or atrial tachycardia lasting ≥30 s occurring within 3 months post-radiofrequency ablation, consistent with the consensus definition (12). Recurrence was assessed using a standardized rhythm monitoring protocol that included scheduled 12-lead electrocardiograms at each follow-up visit (1, 2, and 3 months post-ablation), 24 h Holter monitoring at 1 and 3 months post-ablation, and symptom-driven event recordings at any time. Patients presenting with symptoms suggestive of arrhythmia recurrence between scheduled visits were instructed to seek immediate ECG documentation.

Exclusion criteria included: (1) thrombus formation within the left atrium or left atrial appendage detected by transoesophageal echocardiography (TOE); (2) history of cardiac radiofrequency ablation or other cardiac surgery; (3) severe concomitant conditions including active infection, decompensated heart failure, hepatic or renal dysfunction, or malignancy; (4) uncontrolled thyroid dysfunction; (5) pregnant or lactating women; (6) incomplete clinical documentation.

### 2.4 Procedural approach

All patients underwent preoperative electrocardiogram (ECG), chest x-ray, and coagulation function tests. Transthoracic echocardiography (TTE) was performed to measure left atrial diameter (LAD) and left ventricular ejection fraction (LVEF), while transoesophageal echocardiography (TOE) was used to exclude left atrial and left atrial appendage thrombus prior to the procedure. Multislice spiral computed tomography with three-dimensional reconstruction was used to visualize the pulmonary veins entering the left atrium.

Regarding the periprocedural anticoagulation strategy, all patients receiving oral anticoagulation therapy prior to the procedure continued their regimen in an uninterrupted fashion, consistent with contemporary guideline recommendations (13). For patients not previously anticoagulated, subcutaneous low-molecular-weight heparin was administered for 5–7 days before the procedure. Antiarrhythmic medications were discontinued for at least 5 half-lives prior to surgery. Informed consent was obtained preoperatively.

Radiofrequency ablation was performed by the same experienced team under general anesthesia. The procedure was conducted as follows: a coronary sinus electrode was placed via left subclavian vein puncture. Two transseptal punctures were made via the right femoral vein to advance two SL1 Swartz sheaths. Initial intravenous heparin 100 U/kg was administered, followed by continuous infusion at 1,000 U/h, with activated clotting time monitored every 20–30 min to maintain a target above 300 s. Selective angiography identified the left and right pulmonary veins. The pulmonary vein loop mapping catheter (Lasso) and irrigated-tip ablation catheter (Navi-Star-Cool) were advanced through the sheaths into the left atrium. Under the three-dimensional electroanatomical mapping system (CARTO), the irrigated-tip catheter was used to acquire geometry points in the left atrium, constructing a three-dimensional model of the left atrial–pulmonary vein complex. Circumferential ablation was performed around the pulmonary veins at their antral regions. The Lasso electrode confirmed electrical isolation between the left atrium and pulmonary veins. Residual pulmonary venous potentials were eliminated with additional ablation to achieve complete electrical isolation. For patients with persistent AF, additional ablation was performed at the left atrial roof, mitral isthmus, and sites demonstrating complex fractionated atrial electrograms. Direct-current cardioversion was performed for patients remaining in AF after ablation.

### 2.5 General data collection

General demographic data for both patient groups were retrospectively collected via the electronic medical record system. Key variables included sex, age, body mass index (BMI), smoking history (defined as daily consumption >1 cigarette with >1 year of continuous use or <1 year since cessation), drinking history (defined as daily consumption exceeding 1 drink unit, where 1 unit equals 45 mL of spirits, 360 mL of beer, or 120 mL of fruit wine), hypertension [meeting the diagnostic criteria per the Japanese Society of Hypertension Guidelines for Self-monitoring of Blood Pressure at Home, Second Edition (14)], diabetes [meeting the diagnostic criteria per the Chinese Expert Consensus on Diabetes Classification (15)], hyperlipidemia [meeting the diagnostic criteria per the Japan Atherosclerosis Society Guideline for Diagnosis and Treatment of Hyperlipidemia (16)], AF type, AF duration, AF episode frequency, LAD, and LVEF.

### 2.6 P-Wave electrocardiographic parameters

P-wave electrocardiographic parameters were collected from both patient groups one day prior to surgery using a BeneHeart R12 twelve-channel electrocardiograph. Patients were recorded in a resting supine position during eight to twelve cardiac cycles. Synchronized 12-lead electrocardiography was then performed after verifying ECG acquisition quality, requiring clear waveforms, no significant interference, and a stable baseline. Recordings were obtained at a paper speed of 50 mm/s and a gain of 10 mm/mV. P-wave parameters were manually measured from digital ECG recordings by two experienced cardiologists who were blinded to patient group allocation. The onset and offset of the P-wave were identified as the points of initial and final deviation from the isoelectric line, respectively. Each parameter was measured in at least three consecutive cardiac cycles, and the average value was recorded. The P-wave duration (PWD) for each lead was calculated as the mean of three measured P-waves. The maximum P-wave duration (Pmax) and minimum P-wave duration (Pmin) across all leads were identified, and P-wave dispersion (Pd) was defined as the difference between Pmax and Pmin. Intra-observer variability was assessed by repeated measurements from the same observer at a 2-week interval in a random subset of 30 patients, yielding an intraclass correlation coefficient (ICC) of 0.92 (95% CI: 0.86–0.96). Inter-observer variability between the two cardiologists showed an ICC of 0.89 (95% CI: 0.81–0.94), indicating good measurement reproducibility.

### 2.7 Laboratory indicator collection

Laboratory parameters were collected from both patient groups one day prior to surgery. Venous blood samples (3 mL) were obtained in the morning after an overnight fast. Centrifugation was performed at 2,000 rpm with a 10 cm centrifuge radius for 10 min to separate serum for subsequent testing. BNP was measured using a double-antibody sandwich immunoassay. Albumin (ALB), serum creatinine (Scr), blood urea nitrogen (BUN), and uric acid (UA) were determined via colorimetric methods. Alanine aminotransferase (ALT) and aspartate aminotransferase (AST) were measured using rate methods on the Olympus AU5800 fully automated biochemical analyzer with corresponding reagents (Beckman Coulter, USA).

### 2.8 Statistical methods

Data were analyzed using SPSS 28.0 statistical software. Categorical data were expressed as counts (n) and analyzed using the chi-square test. Continuous data conforming to a normal distribution were expressed as mean ± standard deviation (x¯ ± s), and comparisons between groups were performed using independent-samples *t*-tests. Variables showing statistically significant differences in univariate analysis were included in multivariate logistic regression analysis. A nomogram was constructed based on the logistic regression model. Model apparent performance in the derivation cohort was assessed using receiver operating characteristic (ROC) curves, and the area under the curve (AUC) was calculated. Model calibration was assessed using the Hosmer-Lemeshow goodness-of-fit test and a calibration plot comparing predicted probabilities against observed recurrence rates across decile risk groups. An optimal risk threshold was determined by maximizing the Youden index. Because the same cohort was used for model development and performance assessment, these analyses were interpreted as derivation-cohort apparent performance rather than formal internal validation; no bootstrap optimism correction or split-sample validation was performed. The significance level was set at *α* = 0.05.

## 3 Results

### 3.1 Comparison of general characteristics between recurrence and Non-recurrence groups

No statistically significant differences were observed between the recurrence and non-recurrence groups in terms of sex, age, BMI, smoking history, drinking history, hypertension, diabetes, hyperlipidemia, AF duration, AF episode frequency, LVEF, Pmin, ALB, Scr, BUN, UA, ALT, and AST levels (*P* > 0.05). The proportions of persistent and paroxysmal AF types in both groups were 50:12 vs. 85:53, LAD was 43.10 ± 4.68 vs. 40.82 ± 4.45 mm, PWD was 140.98 ± 12.45 vs. 132.76 ± 11.84 ms, Pmax was 120.35 ± 10.82 vs. 105.20 ± 11.06 ms, Pd was 53.92 ± 5.43 vs. 38.26 ± 5.62 ms, and BNP was 91.30 ± 10.58 vs. 84.26 ± 10.35 pg/mL. All differences were statistically significant (*P* < 0.05), suggesting that AF type, LAD, PWD, Pmax, Pd, and BNP levels may be associated with recurrence after AF radiofrequency ablation, as shown in Table 1.

**Table 1:** Comparison of general patient characteristics between recurrence and non-recurrence groups.

Index	Project	Recurrence (*n* = 62)	Non-recurrence (*n* = 138)	*χ*²/t value	*P* value
Sex (Male/Female)		42/20	89/49	0.764	0.382
Age (years)		60.56 ± 9.22	60.80 ± 9.05	0.590	0.556
BMI (kg/m²)		22.50 ± 4.52	22.78 ± 4.60	0.400	0.689
Smoking history		10	25	0.117	0.732
Drinking history		4	8	0.032	0.857
Hypertension		5	10	0.041	0.839
Diabetes		4	7	0.157	0.692
Hyperlipidemia		4	10	0.042	0.839
AF type	Persistent	50	85	7.078	0.008
	Paroxysmal	12	53		
AF duration (yrs)		2.42 ± 0.65	2.30 ± 0.72	1.123	0.263
AF frequency (/mo)		6.20 ± 1.45	5.92 ± 1.39	1.300	0.195
LAD (mm)		43.10 ± 4.68	40.82 ± 4.45	3.298	0.001
LVEF (%)		57.80 ± 4.25	58.26 ± 4.36	0.695	0.488
PWD (ms)		140.98 ± 12.45	132.76 ± 11.84	4.469	<0.001
Pmax (ms)		120.35 ± 10.82	105.20 ± 11.06	9.019	<0.001
Pmin (ms)		66.50 ± 8.76	65.96 ± 8.62	0.408	0.684
Pd (ms)		53.92 ± 5.43	38.26 ± 5.62	18.415	<0.001
BNP (pg/mL)		91.30 ± 10.58	84.26 ± 10.35	4.418	<0.001
ALB (g/L)		36.25 ± 5.72	35.84 ± 5.80	0.464	0.643
Scr (*μ*mol/L)		67.95 ± 9.26	68.40 ± 9.35	0.316	0.753
BUN (mmol/L)		10.25 ± 2.45	10.47 ± 2.50	0.579	0.563
UA (μmol/L)		352.80 ± 46.28	350.74 ± 45.76	0.293	0.770
ALT (U/L)		60.35 ± 7.22	59.84 ± 7.04	0.470	0.639
AST (U/L)		46.33 ± 6.24	45.90 ± 3.10	0.651	0.516

### 3.2 Assignment table for study variables

AF recurrence after radiofrequency ablation was designated as the dependent variable, with AF type, LAD, PWD, Pmax, Pd, and BNP serving as independent variables for assignment, as shown in Table 2.

**Table 2:** Assignment table for research variables.

Index	Variable type	Assignment method
Postoperative recurrence	Dependent variable	0 = no recurrence, 1 = recurrence
AF type	Independent variable	0 = paroxysmal, 1 = persistent
LAD	Independent variable	Measured value
PWD	Independent variable	Measured value
Pmax	Independent variable	Measured value
Pd	Independent variable	Measured value
BNP	Independent variable	Measured value

### 3.3 Collinearity analysis results

Collinearity diagnosis revealed that none of the six independent variables—AF type, LAD, PWD, Pmax, Pd, and BNP—exhibited variance inflation factors exceeding 5 (range: 1.040–1.321). This indicates the absence of multicollinearity, and all variables were included in the logistic regression model for further analysis (Table 3).

**Table 3:** Results of collinearity analysis.

Index	VIF	Tolerance
AF type	1.040	0.962
LAD	1.099	0.910
PWD	1.129	0.886
Pmax	1.273	0.785
Pd	1.321	0.757
BNP	1.061	0.943

### 3.4 Logistic regression analysis of recurrence after AF radiofrequency ablation

Multivariate logistic regression analysis revealed that AF type, LAD, PWD, Pmax, Pd, and BNP were all independent risk factors for recurrence after AF radiofrequency ablation (OR = 2.598, 1.118, 1.059, 1.133, 1.642, and 1.071, respectively; all *P* < 0.05), as shown in Table 4.

**Table 4:** Logistic regression analysis of recurrence after AF radiofrequency ablation.

Index	*β*	SE	Wald χ²	*P* value	OR	95% CI
AF type	0.955	0.366	6.804	0.009	2.598	1.268–5.323
LAD	0.111	0.036	9.666	0.002	1.118	1.042–1.199
PWD	0.057	0.014	16.605	<0.001	1.059	1.030–1.088
Pmax	0.125	0.020	40.599	<0.001	1.133	1.090–1.177
Pd	0.496	0.083	35.423	<0.001	1.642	1.394–1.933
BNP	0.069	0.017	16.623	<0.001	1.071	1.036–1.107

### 3.5 Construction and derivation-cohort performance of the nomogram model

**Figure 1:**
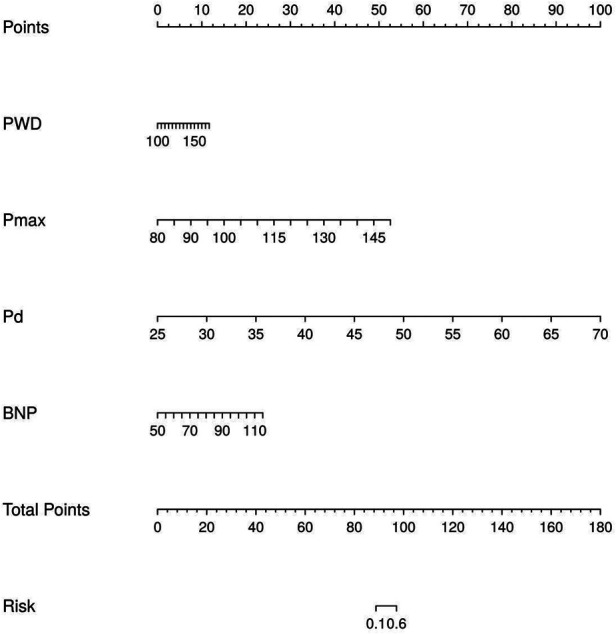
Nomogram model for predicting the risk of recurrence after AF radiofrequency ablation.

Based on the logistic regression results, a nomogram risk prediction model for post-AF radiofrequency ablation recurrence was constructed, incorporating P-wave ECG indicators combined with serum BNP (Figure 1). Each predictor was assigned a score on a 0–100 point scale according to its regression coefficient. The total score, obtained by summing the individual predictor scores, corresponds to a predicted recurrence probability ranging from 0.1 to 0.9. In the present cohort, the total nomogram scores ranged from 42 to 168 points. The median total score was 72 points (interquartile range: 58–98) in the non-recurrence group and 112 points (interquartile range: 95–132) in the recurrence group. By maximizing the Youden index on the ROC curve, an optimal threshold of 85 total points was identified for distinguishing low-risk (≤85 points, predicted recurrence probability <0.35) from medium-to-high-risk (>85 points, predicted recurrence probability ≥0.35) patients. At this threshold, the model achieved a sensitivity of 88.7% and specificity of 84.1%.

**Figure 2:**
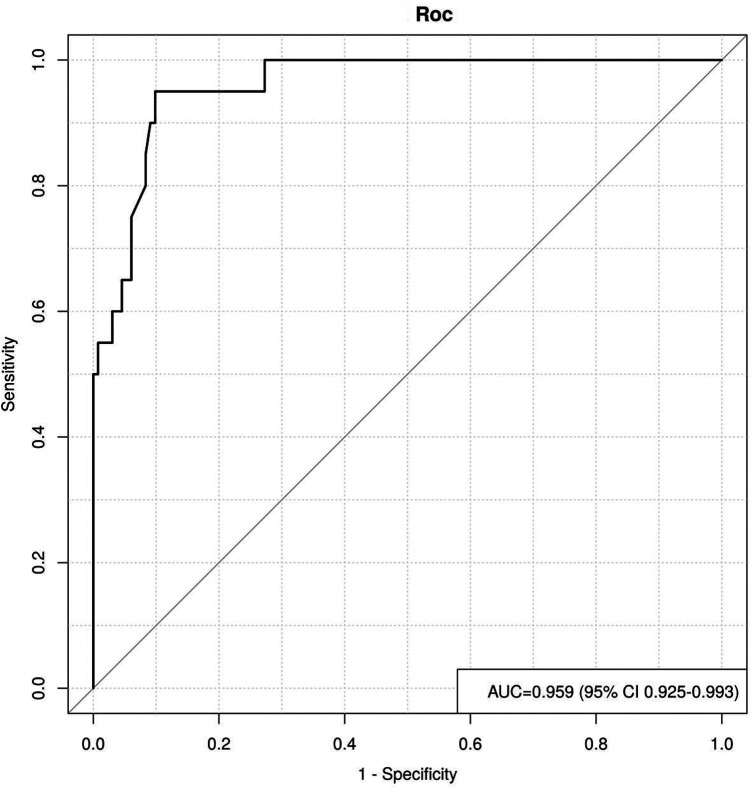
ROC curve showing derivation-cohort apparent performance of the recurrence risk prediction model following AF radiofrequency ablation.

**Figure 3:**
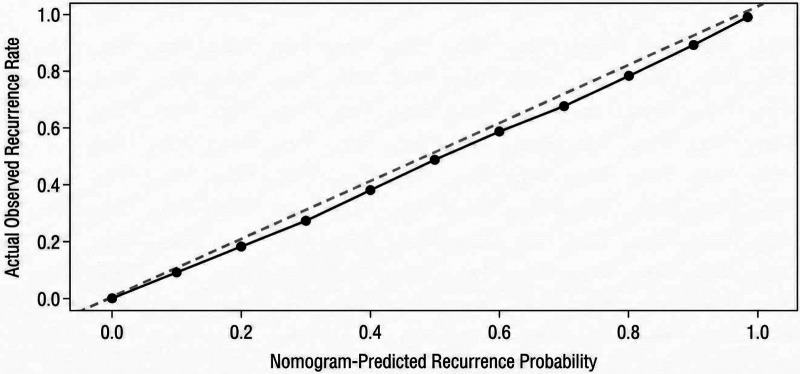
Calibration plot of the nomogram model for predicting AF recurrence after radiofrequency ablation.

The ROC curve describing derivation-cohort apparent performance demonstrated an area under the curve (AUC) of 0.959 (95% CI: 0.925–0.993), indicating excellent discrimination within the model-development dataset (Figure 2). To further evaluate apparent calibration, the Hosmer-Lemeshow goodness-of-fit test was performed, yielding a chi-square value of 5.28 (*P* = 0.726), indicating no significant departure from perfect calibration. A calibration plot was generated by dividing patients into decile groups of predicted risk and plotting the predicted recurrence probability against the observed recurrence rate within each group (Figure 3). The calibration plot demonstrated that predicted probabilities closely aligned with observed recurrence rates across the full range of predicted risk, with the calibration curve closely approximating the 45-degree ideal line. However, because no resampling-based or split-sample procedure was used, these findings should be interpreted as apparent performance in the derivation cohort rather than as formal internal validation.

## 4 Discussion

In the present study, we identified AF type, LAD, PWD, Pmax, Pd, and BNP as independent risk factors for early recurrence after AF radiofrequency ablation in a retrospective cohort of 200 patients. The risk prediction nomogram model integrating these P-wave ECG indicators with serum BNP yielded an AUC of 0.959 (95% CI: 0.925–0.993), with acceptable apparent calibration confirmed by the Hosmer-Lemeshow test (*P* = 0.726) and the calibration plot. Because model performance was assessed in the same dataset used for model development, the reported discrimination and calibration should be regarded as derivation-cohort apparent performance, and the model should be considered a preliminary clinical tool pending formal internal and external validation (17–19).

The finding that AF type and LAD are risk factors for recurrence after radiofrequency ablation is consistent with prior literature. McCabe et al. (20) demonstrated that patients with paroxysmal AF had lower rates of postoperative recurrence compared with those with persistent AF. Persistent AF, characterized by prolonged duration, is associated with more advanced ion channel remodeling, altered excitation-contraction coupling, and conduction abnormalities. These pathological changes increase disease complexity, making it challenging to eliminate all potential triggers and maintenance mechanisms during ablation, thereby elevating recurrence risk (21, 22). Similarly, Cherian et al. (23) reported that the risk of AF recurrence increases with greater LAD, further supporting our findings. Enlarged left atrial diameter often reflects significant alterations in left atrial pressure, ejection fraction, and structural remodeling, including myocardial fibrosis, which can increase myocardial excitability and automaticity, contributing to recurrence (24, 25).

Another important finding was that PWD, Pmax, and Pd served as independent risk factors for recurrence following AF radiofrequency ablation. Electrocardiography remains the most readily accessible clinical tool for diagnosing AF and evaluating ablation outcomes (26). The P-wave, generated by changes in atrial electrical potential, reflects the electrical conduction patterns of the atria and holds significance for assessing AF recurrence risk (27). Research by Boyalla et al. (28) demonstrated that ECG P-wave parameters have significant value for predicting postoperative AF recurrence, with all P-wave parameters showing elevated values in the recurrence group compared to the non-recurrence group, consistent with the present findings. Patients with higher preoperative PWD, Pmax, and Pd exhibit abnormal atrial structure and electrical activity, which reduces conduction velocity, prolongs atrial conduction time, and increases susceptibility to intra-atrial conduction block, thereby elevating recurrence risk (8). From a pathophysiological perspective, prolongation of P-wave duration and increased P-wave dispersion reflect heterogeneous atrial conduction resulting from progressive interstitial fibrosis, myocyte hypertrophy, and gap junction remodeling within the atrial myocardium (29). These structural changes disrupt the uniformity of electrical propagation across the atria, creating areas of slow conduction and functional conduction block that serve as substrates for reentrant circuits (30). It should also be noted that, in addition to the P-wave parameters evaluated in the present study, other ECG-derived markers of atrial remodeling have been reported in the literature. For example, the P-wave terminal force in lead V1 (PTFV1), which reflects left atrial conduction delay and pressure overload, has been identified as a predictor of AF recurrence after catheter ablation in recent studies (31). Similarly, P-wave late potentials detected by signal-averaged ECG have been proposed as indicators of fragmented atrial conduction and atrial substrate modification (29, 30). Although these markers were not assessed in the present study, their incorporation into future integrated risk-prediction models may further enhance the precision of post-ablation recurrence stratification. Therefore, clinical monitoring of ECG parameters in AF radiofrequency ablation candidates is essential. Patients with elevated preoperative P-wave indices may benefit from more intensive pharmacotherapy to optimize heart rate and blood pressure control, reduce left atrial pressure, and delay or reverse left atrial remodeling. In certain cases, a wider antral isolation strategy or additional substrate modification may be considered.

This study also identified BNP as a risk factor for recurrence following radiofrequency ablation for AF. Okada et al. (32) investigated the correlation between BNP levels and postoperative recurrence, concluding that BNP is an independent predictor—a finding consistent with our results. BNP, a neurohormone synthesized and secreted by ventricular cardiomyocytes, serves as a biomarker for heart failure and is also employed to assess prognosis in AF patients (32). Its secretion increases following elevated atrial volume and wall tension, particularly in patients with atrial myocardial hypertrophy. Elevated BNP levels may reflect underlying atrial substrate abnormalities that promote pulmonary vein ectopy and post-ablation atrial fibrosis, thereby increasing recurrence risk (33, 34). Therefore, preoperative assessment of serum BNP levels is clinically warranted in AF patients undergoing ablation. Elevated BNP levels may indicate the need for optimization of heart failure therapies, including angiotensin-converting enzyme inhibitors, angiotensin II receptor blockers, beta-blockers, and diuretics, to improve left ventricular function and indirectly reduce left atrial pressure prior to the procedure.

The risk prediction nomogram constructed in this study has potential clinical applications beyond recurrence prediction. Notably, it may assist clinicians in guiding decisions regarding long-term continuation of anticoagulation after ablation in patients identified as medium-to-high risk. Current international guidelines, including the 2024 European Society of Cardiology (ESC) guidelines for AF management (35), recommend that anticoagulation decisions after ablation should be based on the individual patient's stroke risk profile rather than on procedural success alone. The identification of high-risk patients through our nomogram could prompt more vigilant rhythm surveillance and inform discussions regarding the continuation of oral anticoagulation therapy, particularly in patients with additional thromboembolic risk factors. It should be noted that the dosing of non-vitamin K antagonist oral anticoagulants (NOACs) is determined by specific patient characteristics—including renal function, hepatic function, age, body weight, and potential drug–drug interactions—rather than by thromboembolic risk scores such as CHA^2^DS^2^-VASc (35).

Based on the nomogram risk stratification, we propose the following guideline-aligned continuity of care strategies. For individualized health education, patients in the low-risk group (≤85 points) receive education on postoperative daily precautions, medication adherence, and baseline symptom recognition, while medium-to-high-risk patients (>85 points) additionally receive education on AF recurrence warning signs, the necessity of anticoagulation therapy, and bleeding risk awareness. Follow-up care should be structured in a tiered manner consistent with the CARE-AF approach and the 2024 ESC AF guidelines (35), with low-risk patients undergoing outpatient follow-up at 1, 3, and 6 months post-ablation (including 12-lead ECG and serum BNP assessment), and medium-to-high-risk patients undergoing more intensive monitoring at 1, 2, 3, and 6 months with the addition of 24 h Holter monitoring and echocardiography. Remote rhythm monitoring through commercially available wearable devices capable of detecting AF episodes may be considered to supplement in-clinic assessments, particularly for patients at higher predicted risk. Psychological well-being should be assessed periodically using validated instruments such as the Patient Health Questionnaire-9 (PHQ-9) and Generalized Anxiety Disorder-7 (GAD-7), with patients scoring ≥10 referred for specialized psychological or psychiatric evaluation. Lifestyle interventions should include dietary sodium restriction (<2 g/day), adequate potassium intake, and exercise prescriptions tailored to risk level: moderate-intensity aerobic exercise (150 min per week) for low-risk patients, and low-intensity activities for medium-to-high-risk patients, avoiding maneuvers that may trigger arrhythmias. The total recommended intervention duration is 6 months.

The innovation of this study lies in establishing a risk prediction nomogram model that combines P-wave ECG indicators with serum BNP levels as predictors, enabling visual and quantitative estimation of postoperative early recurrence risk. In the derivation cohort, the nomogram achieved an AUC of 0.959 with acceptable apparent calibration. The identification of an optimal risk threshold at 85 total points provides clinically interpretable criteria for differentiating low-risk from medium-to-high-risk patients, thereby facilitating targeted follow-up and intervention planning. Nevertheless, the performance estimates reported herein are likely to be optimistic to some extent because formal internal validation was not undertaken.

However, this study has several limitations that warrant acknowledgment. First, as a retrospective, single-center study of 200 AF patients, the findings may have limited generalizability to broader populations. Second, the primary endpoint was early recurrence within 3 months post-ablation. Although early recurrence is a clinically relevant outcome, it should be recognized that early recurrence may in part reflect transient post-procedural inflammatory responses and does not uniformly predict late or very late recurrence (36). Our model therefore specifically predicts early recurrence risk, and its applicability to long-term recurrence prediction requires further validation with extended follow-up. Third, P-wave parameters were measured manually from digital ECG recordings, which inherently introduces observer variability despite the good intra- and inter-observer reproducibility reported in our study. Automated P-wave measurement tools may further enhance reproducibility in future studies. Fourth, despite employing a standardized rhythm monitoring protocol including scheduled ECGs and Holter monitoring, the detection of asymptomatic recurrence episodes may have been incomplete, representing a potential source of detection bias. Continuous rhythm monitoring with implantable loop recorders would provide more accurate recurrence ascertainment but was not feasible in this retrospective setting. Fifth, the logistic regression model considered only a limited set of potential predictors, and other factors—such as left atrial fibrosis burden, procedural variables, and genetic predisposition—were not assessed. Sixth, the model was evaluated only in the derivation cohort, without bootstrap optimism correction, cross-validation, or split-sample testing; accordingly, some degree of overfitting cannot be excluded and the reported AUC and calibration may overestimate performance in new patients. Future prospective, multicenter studies with larger sample sizes, longer follow-up periods, formal internal validation, and external validation cohorts are warranted to confirm and extend these findings.

## 5 Conclusions

In this retrospective cohort study, AF type, LAD, PWD, Pmax, Pd, and BNP were identified as independent risk factors for early recurrence after AF radiofrequency ablation. The derivation-cohort nomogram model, constructed using P-wave ECG indicators combined with serum BNP, demonstrated excellent apparent discrimination (AUC = 0.959) and acceptable apparent calibration for predicting early recurrence. Risk stratification using an optimal threshold of 85 total points effectively distinguished low-risk from medium-to-high-risk patients. Clinicians may use this model as a preliminary risk stratification tool when developing personalized follow-up care strategies, although formal internal and external validation remain necessary before broader clinical implementation.

## Data Availability

The original contributions presented in the study are included in the article/Supplementary Material, further inquiries can be directed to the corresponding authors.
